# Hospital Anxiety and Depression Scale (HADS): validation in a Greek general hospital sample

**DOI:** 10.1186/1744-859X-7-4

**Published:** 2008-03-06

**Authors:** Ioannis Michopoulos, Athanasios Douzenis, Christina Kalkavoura, Christos Christodoulou, Panayiota Michalopoulou, Georgia Kalemi, Katerina Fineti, Paulos Patapis, Konstantinos Protopapas, Lefteris Lykouras

**Affiliations:** 1 Second Department of Psychiatry, Athens University Medical School, 'Attikon' General Hospital, Athens, Greece; 2 Third Department of Surgery, University of Athens, School of Medicine, 'Attikon' University Hospital, Athens, Greece; 3 Fourth Department of Internal Medicine, University General Hospital 'Attikon', Athens, Greece

## Abstract

**Background:**

The Hospital Anxiety and Depression Scale (HADS) has been used in several languages to assess anxiety and depression in general hospital patients with good results.

**Methods:**

The HADS was administered to 521 participants (275 controls and 246 inpatients and outpatients of the Internal Medicine and Surgical Departments in 'Attikon' General Hospital in Athens). The Beck Depression Inventory (BDI) and the State-Trait Anxiety Inventory (STAI) were used as 'gold standards' for depression and anxiety respectively.

**Results:**

The HADS presented high internal consistency; Cronbach's α cofficient was 0.884 (0.829 for anxiety and 0.840 for depression) and stability (test-retest intraclass correlation coefficient 0.944). Factor analysis showed a two-factor structure. The HADS showed high concurrent validity; the correlations of the scale and its subscales with the BDI and the STAI were high (0.722 – 0.749).

**Conclusion:**

The Greek version of HADS showed good psychometric properties and could serve as a useful tool for clinicians to assess anxiety and depression in general hospital patients.

## Background

The Hospital Anxiety and Depression Scale (HADS) was developed by Zigmond and Snaith [1] in 1983. Its purpose is to provide clinicians with an acceptable, reliable, valid and easy to use practical tool for identifying and quantifying depression and anxiety. The role of the scale is dimensional rather than categorical; it is best used not to make diagnoses of psychiatric disorders, but for identifying general hospital patients who need further psychiatric evaluation and assistance [2].

Depression and anxiety among general hospital patients could be much higher than is generally assumed, compounding the basic medical condition prognosis. The prevalence of depression in medical and surgical inpatients in Greece, using the Beck Depression Inventory (BDI) [3], was found to be 29% [4]. Proportional findings also include cancer patients (20–25%) [5]. Not only general hospital patients, but also cancer patients have reported that they might benefit from specific interventions aimed at psychological symptoms [6].

The HADS has been translated and widely used in more than 25 countries since its original development [2]. Herrmann, in an extended review, reported that the HADS has demonstrated reliability and validity when used to assess medical patients [2]. Bjelland reached similar conclusions in his review 5 years later [7]. The HADS has been used in the general population [8-10], on general hospital patients [11-14], in cancer care settings [15-17], and even in HIV patients [18]. The HADS has been translated into Greek and validated in a palliative care unit for cancer patients with good results [19].

The aim of this study was to validate the Greek translation of the HADS, and assess its psychometric properties in general hospital patients.

## Methods

### Subjects

The study was performed by the Second Department of Psychiatry at the 'Attikon' General Hospital in Athens. Three groups participated; one group of elderly inpatients in the Internal Medicine and Surgical Departments, one group of outpatients waiting to be examined in the Internal Medicine Outpatients Department and one general population group which was assessed by mail (this group is referred to as 'controls'). A total of 521 participants completed the study; 246 patients and 275 controls.

### Instruments

The HADS is a self-report rating scale of 14 items on a 4-point Likert scale (range 0–3). It is designed to measure anxiety and depression (7 items for each subscale). The total score is the sum of the 14 items, and for each subscale the score is the sum of the respective seven items (ranging from 0–21). It is worth noting that items referring to depression symptoms that describe somatic aspects of depression (e.g. insomnia and weight loss) are not included in the scale. The Greek translation by 'nFer Nelson Publishing' (The Chiswick Centre, 414 Chiswick High Road, London, UK) was used with permission.

The Beck Depression Inventory (BDI) was used to measure depression. It is designed to examine both somatic and cognitive aspects of depression. The BDI is a 21-item self-reporting scale that has been used, apart from its original purpose (assessment of the severity of known depression), for screening purposes. The Greek version has been translated and validated previously [20] and has been widely used to date.

The State-Trait Anxiety Inventory (STAI)[21] developed by Spielberger is used to measure anxiety. It is a 40-item scale made up of two 20-item subscales (one state and one trait), and has been widely used to asses anxiety not only in clinical but in non-clinical samples. The STAI (Form X) has been translated and validated in Greek [22]. The BDI and STAI were administered to patients only.

All of the scales used are self-rated and were administered by five of the researchers. The aim was that the examiners would interfere as little as possible in the patient's completion of the scales. For homogeneity of the results, the scoring of the scales was performed by only one of the researchers.

### Statistical analysis

The following tests were used for the statistical analysis of the data: the Pearson Chi-square test was used for comparison of percentages, and thee Student t test and one-way analysis of variance (ANOVA, with Bonferroni correction) for comparison of means of variables. Correlations were tested by the Pearson r or the Spearman r_s _coefficients, depending on whether the variables were normally distributed or not. The psychometric properties of the HADS were evaluated by the following: construct validity was assessed by inter-item and inter-scale correlations and exploratory factor analysis (principal components with varimax rotation). The intraclass correlation coefficient was used to explore the test-retest reliability. The internal consistency of the scale was calculated with Cronbach's alpha coefficient (minimum acceptable value for alpha was 0.7). Concurrent validity was assessed by calculating correlations between the HADS and the BDI (the gold standard for depression) and the STAI (the gold standard for anxiety). Statistical analysis was carried out using SPSS (Version 11.0) for Windows (SPSS Inc., Chicago, IL, USA).

## Results

### Patient characteristics

The demographic data for the participants are listed in Table 1. The patients group consisted of two subgroups: 150 inpatients (elderly inpatients of age > 65 years attending the Internal Medicine and Surgical Departments) and 96 outpatients of all ages waiting to be examined in the Internal Medicine Outpatients Department. The differences in age and sex among the three groups were of statistical significance.

**Table 1:** Participant emographic data

**Group**	**Number**	**Age (SD)**	**Sex (% male)**
Patients	246	64.68 (17.15)	46.3
Inpatients	150	74.14 (7.21)*	54.7 †
Outpatients	96	49.90 (17.74)*	33.3 †
Controls	275	37.11 (7.62)*	59.3 †

Total	521	50.13 (18.94)	53.2

ANOVA, analysis of variance; SD, standard deviation.

*p < 0.01 by ANOVA, **p < 0.01 by Pearson Chi-squared.

The mean scores for HADS, BDI and STAI are listed in Table 2. Comparing patients as a whole to the controls (t test) showed that patients had greater values as assessed by HADS, HADS-D (depression) and HADS-A (anxiety) with a level of statistical significance p < 0.001. The same finding was generally observed when inpatients and outpatients were compared to controls separately and to each other (using ANOVA after Bonferroni correction); inpatients and outpatients showed higher scores than controls. It is worth noting that although inpatients and outpatients had similar scores on the HADS-D, outpatients showed higher scores on the HADS-A.

**Table 2:** Patient psychometric data

**Group**	**HADS**	**HADS depression**	**HADS anxiety**	**BDI**	**STAI state**	**STAI trait**
Patients (Inpatients + Outpatients)	14.0 (7.9)*	7.3 (4.4)*	6.6 (4.5)*	12.3 (8.4)	43.1 (12.7)	38.5 (11.6)
Inpatients	12.6 (7.9)†	7.3 (4.7)	5.2 (4.2)	10.8 (7.2)	40.4 (12.3)	34.0 (9.4)
Outpatients	16.1 (7.4)†	7.3 (3.8)	8.7 (4.3)†	14.7 (9.5)	48.0 (12.1)	46.7 (10.9)
Controls	9.1 (6.1)*†	3.9 (3.1)*†	5.1 (3.7)*			

Total	11.4 (7.4)	5.5 (4.1)	5.8 (4.2)			

ANOVA, analysis of variance; BDI, Beck Depression Inventory; HADS, Hospital Anxiety and Depression Scale; STAI, State-Trait Anxiety Inventory.

*p < 0.001 in t test patients vs controls; † p < 0.001 in ANOVA inpatients vs outpatients vs controls.

Zigmond and Snaith [1] have suggested two cut-off scores for detecting depression and anxiety that have generally been used in most studies; scores of 8 to 10 = doubtful cases, and scores of 11 and higher = valid cases. Bjelland *et al*., in their review, report that most studies conclude the cut-off score of 8 in general population and in somatic patients samples is correct [7]. The same score has been recently proposed by Olsson *et al*. for outpatients [23]. In our patient sample (inpatients and outpatients), the prevalence of doubtful cases was 14.2% for depression and 16.3% for anxiety. The prevalence of valid cases was 13.4% for depression and 15.1% for anxiety. These percentages for patients only were 22.3% for doubtful cases for depression and 17.4% for anxiety and 22.7% for valid cases for depression and 21.9% for anxiety.

### Psychometric properties of HADS

#### Internal consistency

The HADS Cronbach's α value for the total HADS was 0.884, for anxiety 0.829 and for depression 0.840. Construct validity measured by item-scale correlations ranged from 0.540 to 0.804 and were always higher for each item with its factor (anxiety or depression). For details see Table 3.

**Table 3:** Item-scale correlations and Cronbach's α value

**HADS items**	**HADS**	**HADS depression**	**HADS anxiety**	**Cronbach's α (if item deleted) for the total HADS**
1 (anxiety)	0.696	0.521	**0.719**	0.873
2 (depression)	0.661	**0.748**	0.434	0.875
3 (anxiety)	0.625	0.372	**0.743**	0.877
4 (depression)	0.691	**0.765**	0.469	0.873
5 (anxiety)	0.699	0.484	**0.762**	0.873
6 (depression)	0.755	**0.804**	0.544	0.870
7 (anxiety)	0.645	0.459	**0.687**	0.875
8 (depression)	0.679	**0.720**	0.493	0.874
9 (anxiety)	0.688	0.521	**0.705**	0.873
10 (depression)	0.519	**0.665**	0.261	0.884
11 (anxiety)	0.462	0.215	**0.608**	0.885
12 (depression)	0.669	**0.782**	0.415	0.874
13 (anxiety)	0.582	0.333	**0.702**	0.878
14 (depression)	0.513	**0.540**	0.375	0.882

HADS, Hospital Anxiety and Depression Scale, bold: greater values in item-scale correlations.

#### Test-retest reliability

Fifty of the controls, randomly selected, completed the HADS on two occasions with a 20-day interval. Both the total scale and the two subscales showed high retest stability. The intraclass correlation coefficient for the total HADS was 0.944, for the HADS/anxiety 0.899 and for the HADS/depression 0.837. None of the scales showed statistically significant differences between test and retest.

#### Factor analysis

The HADS has a two-factor structure; factor I for depression and factor II for anxiety. All items, with the exception of item 14 (detecting depression), showed higher scores for the factor they were expected to. The factor loadings are shown in Table 4.

**Table 4:** Factor loadings (n = 521)

**HADS items**	**Factor 1 (depression)**	**Factor 2 (anxiety)**
1 (anxiety)	0.466	**0.528**
2 (depression)	**0.742**	0.188
3 (anxiety)	0.192	**0.712**
4 (depression)	**0.725**	0.245
5 (anxiety)	0.365	**0.645**
6 (depression)	**0.761**	0.307
7 (anxiety)	0.311	**0.623**
8 (depression)	**0.679**	0.275
9 (anxiety)	0.440	**0.547**
10 (depression)	**0.702**	-3.79E-02
11 (anxiety)	-5.17E-02	**0.706**
12 (depression)	**0.752**	0.166
13 (anxiety)	7.875E-02	**0.772**
14 (depression)	0.346	**0.360**

Extraction method: principal component analysis; rotation method: varimax with Kaiser normalisation. HADS, Hospital Anxiety and Depression Scale, bold: greater values of factor loadings in every item.

The correlation between the anxiety factor II of the HADS and the STAI (state) was 0.628 (p < 0.001) and the correlation between depression factor I of the HADS and the BDI was 0.661 (p < 0.001). Correlations between the subscales: HADS/anxiety with HADS/depression: 0.559.

#### Concurrent validity

The BDI and STAI were used as gold standards to assess depression and anxiety correspondingly. The correlations between the BDI and STAI and the total HADS were high; BDI: 0.749, STAI (state): 0.758. The correlation between the HADS/anxiety and STAI (state) was 0.774, and between the HADS/depression and BDI: 0.722 (p < 0.001).

## Discussion

In the present study, the HADS was tested on a sample of Greek general hospital patients (inpatients and outpatients), and controls from the community. The HADS appears to have high internal consistency; Cronbach's α value for the total HADS was 0.884.

The Greek version of the HADS seems to be bidimensional; thus, it could be considered that the two subscales of the HADS measure anxiety and depression independently. The HADS and its two subscales showed high correlations with the gold standards that were used to measure depression (BDI) and anxiety (STAI).

As expected, patients in general appeared to be more depressed and anxious than the subjects from the general population (controls). Outpatients seemed to be more affected than inpatients in presenting anxiety. This could be attributed to the outpatients' concern about hearing bad news as a result of their consultation. By contrast, inpatients are in a way settled in the 'safety' of the ward.

The psychometric properties of the G reek version of the HADS are similar with those of other languages [8,9,12-14]. The HADS generally appears to have a high internal consistency; Cronbach's α values ranged from 0.870 to 0.885 for all the items of the scale. The item-subscale correlations were moderate to high; from 0.608 to 0.762 for the anxiety items, and from 0.540 to 0.804 for the depression items. The HADS appears to be bidimensional as in the original study by Zigmond and Snaith [1]. All items but one (item 14) loaded in the appropriate factor. Similar findings for one or two, but not always the same, items have been reported not loading to the appropriate factor in many other studies. Most studies report the two factor structure of the HADS [2,7], though there are some exceptions that have reported one factor [16], or three [10,17,24], or even four factors [17]. The study of Mykletun *et al*. with 51,930 participants, which is the largest of all in the literature, concluded a bidimensional structure for the HADS was correct [9].

The HADS property of consisting of two independent subscales can also be shown by its correlations with the scales that were used as gold standards for depression and anxiety. The HADS/anxiety correlated highly with the STAI and the HADS/depression correlated highly with the BDI. There are some studies where the total HADS showed greater correlations than its subscales with BDI and STAI correspondingly [8,12,16,25], but in our study the total HADS correlated to almost the same levels as its corresponding subscales with BDI and STAI (a little higher than HADS/depression with BDI, and a little lower than HADS/anxiety with STAI). It is worth noting that the two HADS subscales had a moderate correlation (0.559) to each other. This could be expected, bearing in mind that depression and anxiety show great comorbidity, especially in general health care settings [26].

There are some limitations in our study; there were gender and age differences between the groups, and the test-retest reliability was carried out with the control group only.

## Conclusion

The findings of the present study suggest that the Greek version of the HADS is acceptable, reliable and valid. It could be used in general hospitals to assess depression and anxiety, helping clinicians identify patients who need special psychiatric care.

## Authors' contributions

IM was co-designer of the study and drafted the manuscript, AD participated in data collection and drafted the manuscript, CK participated in data collection and processing, CC participated in data collection and revised the manuscript, PM participated in data collection and revised the manuscript, GK was co-designer of the study and participated in data collection, KF participated in data collection and processing, PP participated in data collection and processing, KP participated in data collection and processing, and LL was co-designer of the study and gave final approval to the published version. All authors read and approved the final manuscript.
